# Critical Care Nurses' Attitude Towards Caring for Dying Patients and Its Relationship With Authenticity in Southeastern Iran: A Descriptive Correlational Study

**DOI:** 10.1002/hsr2.71625

**Published:** 2025-12-11

**Authors:** Alireza Alizadeh, Maryam Heidarinejad, Asma Hajalizadeh, Farideh Razban

**Affiliations:** ^1^ Reproductive and Family Health Research Center Kerman University of Medical Sciences Kerman Iran; ^2^ Afzalipour Hospital, Kerman University of Medical Sciences Kerman Iran; ^3^ Clinical Research Development Unit Shahid Bahonar Hospital, Kerman University of Medical Sciences Kerman Iran; ^4^ Nursing Research Center Kerman University of Medical Sciences Kerman Iran

**Keywords:** attitude, authenticity, caring for dying patients, critical care, nurses

## Abstract

**Background and Aims:**

End‐of‐life care in the ICU is essential to relieve suffering and maintain patient dignity during the final stages of life. The attitudes of critical care nurses toward caring for dying patients significantly influence the quality of care provided. Research suggests that higher levels of authenticity are associated with lower death anxiety. This study aimed to examine the relationship between attitudes towards caring for dying patients and its correlation with authenticity among intensive care unit nurses in southeastern Iran. However, this study was not able to determine a cause‐and‐effect relationship between these two variables.

**Methods:**

This descriptive correlational study was conducted in ICUs in Southeast Iran. The attitudes of 142 critical care nurses toward end‐of‐life care were measured using the Frommelt Attitude toward Care of the Dying (FATCOD) scale, while their authenticity was assessed with the Kernis‐Goldman Authenticity Inventory‐Short Form (KGAI‐SF).

**Results:**

Participants' mean age was 34.93 years, and they were mainly female (76.4%), and held a bachelor's degree (86.1%). The total mean score of nurses' attitudes toward caring for dying patients was 98.99 ± 10.59 out of 150, with the lowest scores associated with items reflecting direct exposure to and reminders of patient death. The total mean score of authenticity was 3.44 ± 0.37 out of 5. A significant positive correlation was identified between the total FATCOD score and the total KGAI‐SF score (r = 0.50; *p* < 0.001; 95% CI [0.33, 0.61]) and all its subscales (*p* < 0.05).

**Conclusion:**

For cultivate authenticity, nurses should reflect on their thoughts and behaviors for greater self‐awareness, which can be effectively promoted through mindfulness and meditation courses. Reflecting on their attitudes towards death and end‐of‐life care can also reveal unconscious beliefs affecting their behavior.

## 1 Introduction

Given the critical condition of patients admitted to the intensive care unit (ICU), death in this unit is a common and significant concern [1]. Care of dying patients in the ICU is of particular importance. This includes symptom management, emotional and psychological support, and assurance of patient dignity and respect. The main goal is to reduce patient pain and to ensure a peaceful and comfortable environment for patients during their final days [2]. Studies have shown that end‐of‐life care helps improve the remaining quality of life and reduces anxiety and fear [3].

Nurses play a vital role in the care of dying patients. They must be able to effectively communicate with patients and families and carefully assess and manage the physical and psychological needs of the patient. Their collaborative role within the healthcare team is crucial for delivering optimal end‐of‐life care [4].

Nurses' attitudes toward caring for dying patients can have a significant impact on the quality of care. A positive and empathetic attitude can help improve the patient's remaining quality of life and reassure families [5], while a negative or avoidant attitude can decrease the quality of care and increase anxiety and stress in both patients and families [6].

Negative attitudes toward caring for dying patients often stem from the lack of acceptance of death itself. Many people, including nurses, perceive death as a taboo and are afraid to face it [7]. Greenberg's Terror Management Theory (TMT) posits that the awareness of our own mortality creates a fundamental psychological conflict that leads to anxiety and stress. This conflict arises from the desire to survive and be alive, which clashes with the inescapable reality of death [8]. To cope with this anxiety, humans develop various defense mechanisms, which can be both conscious and unconscious and live in a way that avoids facing death [9]. However, death anxiety can be triggered by the death of others, as it can remind individuals of their own death [10].

Existential philosophers and positive psychologists believe that individuals with higher levels of authenticity have lower levels of death anxiety [11]. Authenticity refers to an honest awareness of the inner truth or true self, the ability to behave and express oneself in accordance with the true self [12]. Authenticity means being true to one's inner self, aligning one's actions and words with his/her values, feelings, and needs [13]. Authenticity involves being true to oneself, processing thoughts without bias, acting on true desires, and having open, honest relationships. Being authentic can lead to greater peace of mind and less anxiety when facing life's challenges, including the fear of death [14]. Authentic individuals face reality rather than running away from it. They stop distorting or mentally censoring the harsh and inevitable realities of life, such as death, and embrace them with courage [12]. Authenticity allows one to recognize death as inevitable, reducing fear and anxiety. This perspective focuses on living well, particularly in the final stages of life and during the loss of loved ones [15]. This acceptance of death can help reduce anxiety and fear of death and make people feel more at peace at the end of their lives and even when facing the death of loved ones [16].

A review of the literature revealed no studies exploring the relationship between authenticity and attitudes toward caring for dying patients. However, two studies in Iran demonstrated an inverse relationship between authenticity and death anxiety in cancer patients [14] and those with acute respiratory tract infections [17]. Also, A study conducted by Seto et al. in the United States found that how vividly individuals recall their own death experiences, as well as the deaths of a closed other, was linked to a higher level of authenticity [11]. Andrew et al., in Australia found that people who were chronically exposed to genuine mortality cues (funeral/cemetery workers) lived more authentic lives [18]. Recognizing the research gap, the present study aimed to investigate the attitude toward caring for dying patients and its correlation with authenticity among intensive care unit nurses in southeastern Iran. However, this study was not able to determine a cause‐and‐effect relationship between these two variables.

## 2 Methods

### 2.1 Procedure

This descriptive correlational study, approved by the Ethics Committee of Kerman University of Medical Sciences (Ethical Code: IR. KMU. REC.1403.311, Tracking Code: 403000421), collected data from October 2024 to February 2025 within the ICUs of three teaching hospitals affiliated with Kerman University of Medical Sciences: Shafa, Afzalipour, and Bahonar. These hospitals comprised five general ICUs, two trauma ICUs, one surgical ICU, and one poisoning ICU. Data collection adhered to the principles outlined in the Declaration of Helsinki. Before participation, nurses received a detailed explanation of the study's objectives, and were assured of their voluntary involvement and the confidentiality of their responses. They were also informed that they had the right to withdraw from the study at any point without facing any repercussions.

### 2.2 Participants

The study included ICU nurses with a minimum of 6 months of experience in these units. Nurses were excluded if they had experienced a severe stressful event (e.g., death or severe illness of a close relative, divorce) or reported acute mental and psychological problems within the 6 months before data collection. Questionnaires that had over 20% of items unanswered were excluded from the analysis. Only one questionnaire met this criterion. A census sampling method was employed, resulting in a final sample of 142 nurses. At the end of the study, post hoc power analysis was carried out using G*Power 3.1.9.7 software (alpha = 0.05, total sample size = 142, effect size = 0.3) and the power was 0.98.

### 2.3 Measures

Background information potentially influencing authenticity levels or attitudes toward caring for dying patients was assessed. This included: (1) demographic information such as age, gender, marital status, and education le vel, and (2) occupational information, including total nursing experience and ICU nursing experience.

The Frommelt Attitude toward Care of the Dying (FATCOD) scale, developed by Frommelt in the United States in the 1990s [19], is a 30‐item instrument assessing nurses' attitudes toward end‐of‐life care. Using a five‐point Likert scale (strongly disagree to strongly agree), the FATCOD comprises 15 positive and 15 negative items, yielding a total score ranging from 30 to 150, with higher scores indicating more positive attitudes. The scale's developer demonstrated its high internal consistency [20, 21], reporting a Pearson correlation coefficient of 0.926 as evidence of good reliability. Content and construct validities were adequate with an inter‐rater agreement of 1.00 [21]. In 2008, Iranmanesh et al. [22] translated the FATCOD into Persian and evaluated its psychometric properties, reporting a content validity of 69% and a Cronbach's alpha of 0.77 for its reliability.

The Kernis‐Goldman Authenticity Inventory‐Short Form (KGAI‐SF) developed by Goldman and Kernis in the United States in 2004 [23] measures authenticity across four domains: awareness, unbiased processing, behavior, and communication. This 20‐item instrument employs a five‐point Likert scale (1 = strongly disagree to 5 = strongly agree). The mean score is calculated by dividing the sum of all item scores by 20, resulting in a final score between 1 and 5, where higher scores indicate greater authenticity. The KGAI‐SF has demonstrated acceptable reliability, with Cronbach's alpha coefficients of 0.72 (awareness), 0.71 (unbiased processing), 0.70 (behavior), 0.81 (communication), and an overall composite alpha of 0.87. Its validity has also been appropriate [24]. As explained below, the researchers of the present study translated and validated this questionnaire.

To the best of our knowledge, the KGAI‐SF had not been previously used in Iran; therefore, it was translated into Persian using a standard forward‐backward translation method. Initially, two native Persian‐speaking translators, one with expertise in authenticity research, independently translated the original English scale into Persian. Subsequently, two independent native English‐speaking translators, blinded to the original questionnaire, back‐translated the Persian version into English. The two back‐translated versions were compared, minor discrepancies were resolved, and necessary corrections were made. To assess content validity, 10 faculty members and experts from the Razi School of Nursing, Kerman, reviewed the translated questionnaires and their feedback was incorporated. The content validity index (CVI) of the KGAI‐SF was 0.94. Reliability was assessed in a pilot study with 25 participants, yielding a Cronbach's alpha of 0.83.

### 2.4 Statistical Analysis

All statistical analyses were conducted using SPSS software (IBM SPSS, V22). Descriptive statistics (absolute and relative frequencies, means, and standard deviations) were used to summarize background information and questionnaire scores. The normality of variables was confirmed using the Kolmogorov‐Smirnov test. Pearson's correlation coefficient was calculated to examine the relationship between the mean FATCOD score and the mean scores obtained from the KGAI‐SF. Multiple regression analysis was employed to determine the association between FATCOD scores and nurses' background information. Variables with a p‐value less than 0.2 in univariate linear regression were entered into the multiple linear regression model using the backward elimination method.

## 3 Results

### 3.1 Background Information

According to Table 1, the results showed that the mean age of the nurses participating in this study was 34.93 ± 8.01 years. Most of the participants were female (76.4%), married (66.7%), and had a bachelor's degree (86.1%). Their total mean nursing experience was 10.54 ± 6.71 years and their mean ICU experience was 6.83 ± 6.20 years.

**Table 1:** Background information of nurses.

Variables	Number (Percent)	Mean ± SD
Age		34.93 ± 8.01
Gender		
Male	34 (23.6)	
Female	110 (76.4)	
Marital status		
Single	48 (33.3)	
Married	96 (66.7)	
Level of education		
Bachelor of science	124 (86.1)	
Master of science	20 (13.9)	
Total years of nursing experience		10.54 ± 6.71
Years of nursing experience in ICU		6.83 ± 6.20

### 3.2 Nurses' Attitudes Toward Caring for Dying Patients

As shown in Table 2, the total mean score of nurses' attitudes toward caring for dying patients was 98.99 ± 10.59 out of 150. The most negative attitudes were related to items “I would be uncomfortable talking about impending death with the dying person” (2.17 ± 0.96), “I would be upset when the dying person gave up hope of getting better” (2.37 ± 0.98), “It is difficult to form a close relationship with the dying patient” (2.38 ± 1.05), “I would be uncomfortable if I entered the room of a terminally ill person and found him/her crying” (2.46 ± 1.07), and “I would hope the person I am caring for dies when I am not present” (2.55 ± 1.09).

**Table 2:** Mean scores for individual items of FATCOD scale.

Items	Mean ± SD
1. Giving care to the dying person is a worthwhile experience.	3.47 ± 1.17
2. Death is not the worst thing that can happen to a person.	3.54 ± 1.18
3. I would be uncomfortable talking about impending death with the dying person.	2.17 ± 0.96
4. Caring for the patient's family should continue throughout the period of grief and bereavement.	3.69 ± 1.10
5. I would not want to care for a dying person.	2.90 ± 1.01
6. The nonfamily caregivers should not be the one to talk about death with the dying person.	3.16 ± 1.04
7. The length of time required giving care to a dying person would frustrate me.	2.74 ± 1.12
8. I would be upset when the dying person I was caring for gave up hope of getting better.	2.37 ± 0.98
9. It is difficult to form a close relationship with the dying person.	2.38 ± 1.05
10. There are times when the dying person welcomes death.	3.46 ± 0.97
11. When a patient asks, “Am I dying?” I think it is best to change the subject to something cheerful.	2.81 ± 1.10
12. The family should be involved in the physical care of the dying person.	3.72 ± 0.99
13. I would hope the person I'm caring for dies when I am not present.	2.55 ± 1.09
14. I am afraid to become friends with a dying person.	3.20 ± 1.12
15. I would feel like running away when the person actually died.	2.97 ± 1.02
16. Families need emotional support to accept the behavior changes of the dying person.	4.08 ± 0.73
17. As a patient nears death, the nonfamily caregiver should withdraw from his/her involvement with the patient.	3.75 ± 1.00
18. Families should be concerned about helping their dying member make the best of his/her remaining life.	4.13 ± 0.96
19. The dying person should not be allowed to make decisions about his/her physical care.	3.65 ± 1.01
20. Families should maintain as normal an environment as possible for their dying member.	3.92 ± 0.90
21. It is beneficial for the dying person to verbalize his/her feelings.	3.92 ± 0.88
22. Care should extend to the family of the dying person.	3.66 ± 1.08
23. Caregivers should permit dying persons to have flexible visiting schedules.	3.71 ± 1.02
24. The dying person and his/her family should be the in‐charge decision‐makers.	3.56 ± 1.00
25. Addiction to pain relieving medication should not be a concern when dealing with a dying person.	3.84 ± 1.02
26. I would be uncomfortable if I entered the room of a terminally ill person and found him/her crying.	2.46 ± 1.07
27. Dying persons should be given honest answers about their condition.	3.43 ± 0.98
28. Educating families about death and dying is not a nonfamily caregiver responsibility.	3.13 ± 1.02
29. Family members who stay close to a dying person often interfere with the professional's job with the patient.	2.91 ± 0.98
30. It is possible for nonfamily caregivers to help patients prepare for death.	3.37 ± 0.99
Total score	98.99 ± 10.59

### 3.3 Nurses' Authenticity

Based on Table 3, the total mean score of authenticity was 3.44 ± 0.37 out of 5. Relational orientation showed the highest mean score (4.03 ± 0.60).

**Table 3:** Mean scores for KGAI‐SF.

Categories	Mean ± SD
Awareness	3.06 ± 0.51
Unbiased processing	3.05 ± 0.55
Behavior	3.63 ± 0.53
Relational Orientation	4.03 ± 0.60
Total mean	3.44 ± 0.37

### 3.4 The Relationship Between Nurses' Attitude Towards Caring for Dying Patients and Their Authenticity

Table 4 indicates a positive and significant relationship between the total FATCOD score and the total KGAI‐SF score (*r* = 0.50, *p* < 0.001, 95% CI [0.33, 0.61]) and all its subscales. This indicates a positive correlation between nurses' authenticity across all dimensions and their positive attitudes toward caring for a dying patient. The strongest correlation was observed with the Behavior subscale (*r* = 0.49, *p* < 0.001, 95% CI [0.23, 0.43]), while the weakest, though still significant, correlation was with the Unbiased Processing subscale (*r* = 0.20, *p* = 0.02, 95% CI [0.02, 0.23]).

**Table 4:** The correlation between FATCOD and KGAI‐SF.

KGAI‐SF	FATCOD (Total score)		
	*r*	*P*	CI
Awareness	0.33	< 0.001	0.12–0.34
Unbiased processing	0.20	0.02	0.02–0.23
Behavior	0.49	< 0.001	0.23–0.43
Relational Orientation	0.35	< 0.001	0.11–0.30
Total score	0.50	< 0.001	0.33–0.61

### 3.5 The Relationship Between Nurses' Attitude Towards Caring for Dying Patients and Their Background Information

Multiple linear regression results, presented in Table 5, revealed that nurses holding a master's degree had a mean FATCOD score of 0.18 points higher than those with a bachelor's degree (*p* = 0.02). Additionally, each 1‐year increase in age was associated with a 0.30‐point decrease in the mean FATCOD score, while each additional year of work experience correlated with a 0.09‐point increase (*p* = 0.002).

**Table 5:** The relationship between FATCOD and background information.

Variable	Univariate			Multivariate		
	Beta	95% CI	*p* value	Beta	95% CI	*p* value
Age	−0.22	−0.51 to −0.07	0.009	−0.30	−0.65 to −0.14	0.002
Gender	−0.06	−5.64 to 2.65	0.47			
Marital status	−0.18	−7.72 to −2.29	0.35			
Level of education	0.15	−0.51 to 9.54	0.07	0.18	0.59 to 10. 53	0.02
Total years of nursing experience	−0.13	−0.47 to 0.05	0.12	0.09	−0.16 to 0.49	0.32
Years of nursing experience in ICU	−0.03	−0.34 to 0.24	0.72			
				Adjusted *R* ^*2*^ *:0.07*		
				*F* for change in *R* ^*2*^ *:4.47 p:0.005*		

Abbreviations: Beta = unstandardized coefficient, CI = Confidence interval.

*p < 0.05; **p < 0.01.

## 4 Discussion

This study aimed to assess ICU nurses' attitudes toward caring for dying patients and their relationship with authenticity. The results showed that the total mean attitude score was 98.99 ± 10.59 out of 150. Consistent with previous research on Iranian [25, 26, 27], Turkish [28, 29], Spanish [30, 31], American [32] and British nurses [33]. This score indicates an above‐average attitude. The lowest scores in our study were associated with items reflecting nurses' direct exposure to and reminders of patient's death. These included being uncomfortable talking about impending death, being upset when the dying person gave up hope of getting better, being difficult to form a close relationship with the dying patient, being uncomfortable when finding a patient crying, and hoping the patient would dies in the nurse's absence.

This finding is consistent with a multicenter study of 569 nursing students across Italy, Spain, and the United Kingdom, which utilized the FATCOD‐B scale and revealed that the most negative attitudes were linked to items indicating feelings of “uncomfortable talking about impending death” and “being upset when the dying person gave up hope of getting better” [34]. This alignment suggests that the reluctance to confront patient death, as observed in our sample, is not unique to the Iranian context but is also a fundamental issue in nursing across Europe.

This avoidance of confronting and thinking about death aligns with existentialism. Heidegger argues that inauthentic individuals evade this reality by immersing themselves in daily routines and conforming to societal norms [35].

The fear of death, as described by May, goes beyond physical loss. It stems from the potential destruction of one's entire self—psychological, spiritual, physical, and even personal identity. Death is perceived as the ultimate threat to meaning and purpose, causing anxiety when remembering it [36]. Jaspers argues that the solace humans find in traditional meanings like family, religion, and art dissolves when confronted with death [37], and the loss of these meanings is deeply distressing [38]. Heidegger believes that death abruptly destroys all senses of familiarity and belonging [39].

Our results revealed a positive and significant correlation between the total FATCOD score and the total KGAI‐SF score, as well as all its dimensions. However, this correlation does not provide information about the cause‐and‐effect relationship between the two variables. This correlation indicates that greater authenticity in nurses—across awareness, unbiased processing, behavior, and communication—was associated with positive attitudes toward caring for dying patients. While no studies directly examined this relationship, Nazari et al. [14] found an inverse correlation between authenticity (in the authenticity inventory total score as well as in the awareness and behavior dimensions) and death anxiety in cancer patients. Similarly, Darban et al. [17] reported an inverse relationship between all dimensions of authenticity inventory and death anxiety in patients with acute respiratory tract infections. It seems that authentic people have lower death anxiety, accept the reality of death, and feel more comfortable when caring for and communicating with dying patients and their families. However, this aspect was not measured in the current study and should be explored in future research.

From an existentialist viewpoint, awareness of death is essential for achieving authentic living. Heidegger, in “Being and Time” (1927) [40], posits that confronting the certainty and inevitability of death liberates us from an inauthentic life marked by societal conformity. Authenticity involves embracing life's challenges, including death and personal responsibility, rather than yielding to social pressures [12]. For Heidegger, life and death are intertwined, and facing death with courage allows individuals to transcend fear and reconsider their priorities [41]. In Islamic and Iranian culture, reflecting on death encourages a focus away from materialism [42]. The saying “Die before you die,” attributed to Prophet Muhammad, signifies a spiritual transformation of the ego or false self, emphasizing the need to abandon the false self to live authentically [43].

Although in the present study, “authenticity” was examined. In the above sentences the results were discussed with respect to the concept of “existential authenticity”. Both concepts of “authenticity” and “existential authenticity” emphasize “honesty with oneself,” but the meaning of “self” in each is different. In the concept of authenticity, honesty with oneself is behaving and expressing oneself in accordance with one's recognized identity and personal characteristics, values, and beliefs. In the concept of existential authenticity, honesty with oneself is not simply accepting and adhering to a declared identity. Rather, it involves the honest and unadulterated acceptance of fundamental truths such as freedom, responsibility, and death. In the concept of existential authenticity, it is broader and deeper [44].

It sould be noted that a more positive attitude towards death and care for dying patients might foster greater authenticity. Existential philosophy and positive psychology suggest that a profound awareness of death prompts individuals to reevaluate their worldview, clarify personal values, and shift their goals accordingly, enhancing authenticity. Although, a weaker, more abstract awareness of death often triggers defensive reactions, resulting in increased conformity to societal norms rather than personal growth [45, 46].

The results showed that the age of the participants was negatively correlated with the FATCOD score. Older nurses had a more negative attitude towards caring for dying patients. This might be related to burnout or cumulative stress from repeated exposure to patient death over a long career, leading to emotional distancing as a coping mechanism. Some previous studies in United States [20, 47], Palestine [48], Sweden [49] and Iran [49] found that the age of nurses was correlated with their attitude towards caring for dying patients, however, unlike the present study, this relationship was mainly positive. For instance, Deffner and Bell [47] in the United States reported that the older age and more experience among nurses were associated with increased comfort in providing care for dying patients. This discrepancy between our findings and previous research may be linked to the possibility that older nurses in our study experience heightened stress and burnout due to limited institutional support. In contrast, their counterparts in other countries may benefit from more effective coping mechanisms or enhanced organizational support. There is a need for further exploration in future cross‐cultural studies.

Education level was significantly associated with FATCOD. Nurses with a master's degree had a more positive attitude towards caring for the dying patient than nurses with a bachelor's degree. Previous studies in Israel [50] and Sweden [51] also reported that higher education level or participation in educational courses related to caring for the dying patient was associated with more positive attitudes in nurses. For instance Deffner and Bell [47] in the United States reported that nurses who had received education on communicating difficult subjects like death reported significantly higher comfort levels when talking with patients and families. Master of Science in Nursing programs typically incorporate specialized courses and practical experiences in areas such as palliative care, end‐of‐life care, ethics, and effective communication. This training helps nurses feel more confident and skilled when dealing with tough situations like talking with patients and families, and meeting the complex needs of dying patients. To better support all nurses in this area, it would be beneficial to include similar training as part of ongoing professional education, particularly for those who frequently care for dying patients, such as critical care nurses.

The total mean authenticity score was 3.44 ± 0.37 out of 5. The Relational Orientation subscale obtained the highest mean score (4.03 ± 0.60). While no studies examined nurses' authenticity, Nazari et al. and Darban et al. [14, 17] reported above‐average authenticity scores in cancer patients and those with acute respiratory tract infections.

## 5 Conclusion

Our results demonstrated a positive relationship between nurses' authenticity and attitudes toward caring for dying patients, highlighting the importance of fostering authenticity. To achieve this, nurses should reflect on their thoughts and behaviors for greater self‐awareness, which can be effectively promoted through mindfulness and meditation courses. At the same time, training sessions can empower nurses to resist societal pressures and embrace their true selves. Reflecting on their attitudes towards death and end‐of‐life care can also reveal unconscious beliefs affecting their behavior. Simulation‐based training for difficult conversations, reflective practice groups where nurses can safely share their experiences and fears could enhance nurses' self‐awareness of their own death‐related fears and promote acceptance. Familiarity with philosophical concepts like existential crisis can also deepen nurses' understanding of their reluctance to confront patients' death. Considering the limitations of current research, future studies should empirically investigate the effects of these and other psychological interventions on the level of nurses' authenticity and attitudes toward caring for dying patients. In view of the significance of individual authenticity, it is crucial to highlight this aspect in nursing education and policymaking.

## 6 Limitation

A limitation of this study is that while the variable under investigation was “authenticity,” the discussion also included the concept of “existential authenticity. While related, the two are not synonymous. Authenticity focuses on acting with inner self‐confidence and autonomy. An authentic individual can ignore existential principles such as the acceptance of death, the value of choice, responsibility, freedom, and existential solitude, while acceptance of existentialism is a necessary condition for existential authenticity. Thus, existential authenticity extends beyond the scope of authenticity [52]. Developing a tool to assess existential authenticity in nurses could help overcome this limitation in future studies. The study relied exclusively on self‐report questionnaires, which may be subject to social desirability bias, where nurses might report more positive attitudes and more authentic than they actually hold. The sample was drawn from ICUs in three hospitals in Southeastern Iran. Therefore, the findings may not be generalizable to nurses in other regions, countries, or different cultural contexts. This cross‐sectional study examined only the relationship between two variables, leaving the cause‐and‐effect unclear regarding whether authenticity leads to a more positive attitude toward caring for dying patients. Future longitudinal or interventional studies are recommended to establish this relationship. Additionally, using more robust statistical methods like SEM and exploring mediating factors such as death anxiety, existential crisis, and self construal could reveal other dimensions of these relationships. Considering the abstract nature of these concepts, conducting qualitative interviews alongside quantitative assessments in future studies could yield more comprehensive insights. Additionally, the relationship between authenticity and FATCOD may be influenced by factors such as age and education level, which should be addressed in future studies.

## Author Contributions

**Alireza Alizadeh:** conceptualization, writing – original draft, methodology, writing – review and editing, software, data curation. **Maryam Heidarinejad:** conceptualization, methodology, resources, data curation. **Asma Hajalizadeh:** conceptualization, methodology, resources, data curation. **Farideh Razban:** writing – original draft, methodology, validation, writing – review and editing, formal analysis, project administration, data curation, supervision, conceptualization, investigation.

## Funding

The authors received no specific funding for this work.

## Consent

All authors have agreed to be so listed and have read and approved the manuscript and its publication on the Health Science Reports.

## Conflicts of Interest

The authors declare no conflicts of interest.

## Transparency Statement

The lead author Farideh Razban affirms that this manuscript is an honest, accurate, and transparent account of the study being reported; that no important aspects of the study have been omitted; and that any discrepancies from the study as planned (and, if relevant, registered) have been explained.

## Data Availability

The authors confirm that the data supporting the findings of this study are available within the article. Additional de‐identified data may be made available upon reasonable request to the corresponding author. All authors have read and approved the final version of the manuscript. First author had full access to all of the data in this study and takes complete responsibility for the integrity of the data and the accuracy of the data analysis.
